# Development of a Wide Area 3D Scanning System with a Rotating Line Laser

**DOI:** 10.3390/s21113885

**Published:** 2021-06-04

**Authors:** Jaeho Lee, Hyunsoo Shin, Sungon Lee

**Affiliations:** 1 Department of Electrical and Electronic Engineering, Hanyang University, Ansan 15588, Korea; pmh5050@hanyang.ac.kr (J.L.); shs2316@hanyang.ac.kr (H.S.); 2 School of Electrical Engineering, Hanyang University, Ansan 15588, Korea

**Keywords:** 3D reconstruction, laser-camera calibration, triangulation

## Abstract

In a 3D scanning system, using a camera and a line laser, it is critical to obtain the exact geometrical relationship between the camera and laser for precise 3D reconstruction. With existing depth cameras, it is difficult to scan a large object or multiple objects in a wide area because only a limited area can be scanned at a time. We developed a 3D scanning system with a rotating line laser and wide-angle camera for large-area reconstruction. To obtain 3D information of an object using a rotating line laser, we must be aware of the plane of the line laser with respect to the camera coordinates at every rotating angle. This is done by estimating the rotation axis during calibration and then by rotating the laser at a predefined angle. Therefore, accurate calibration is crucial for 3D reconstruction. In this study, we propose a calibration method to estimate the geometrical relationship between the rotation axis of the line laser and the camera. Using the proposed method, we could accurately estimate the center of a cone or cylinder shape generated while the line laser was rotating. A simulation study was conducted to evaluate the accuracy of the calibration. In the experiment, we compared the results of the 3D reconstruction using our system and a commercial depth camera. The results show that the precision of our system is approximately 65% higher for plane reconstruction, and the scanning quality is also much better than that of the depth camera.

## 1. Introduction

Today, many application fields require technology for 3D modeling of real-world objects, such as design and construction of augmented reality (AR), quality verification, the restoration of lost CAD data, heritage scanning, surface deformation tracking, 3D reconstruction for object recognition and so on [1,2,3,4,5,6,7]. Among the non-contact scanning methods, the method using a camera and a line laser is widely used because the scanning speed is relatively fast and there is no damage to the object [8,9]. There are two methods for measuring the distance with the laser: the laser triangulation (LT) method and the time-of-flight (ToF) method. The LT method is slower but it has more precise results than the ToF method [10]. However, both methods have relatively small measurable zones. They scan a point or a line, at most, a narrow region at a time. A laser range finder (LRF) was developed to overcome this limitation. It extends the scan area by rotating the light source [11]. The scan area can be further increased by rotating the LRF. Recently, various methods for estimating the rotation axis of the LRF have been studied to obtain a 3D depth map of the environment [12,13,14,15,16,17,18,19,20,21,22].

**Figure 1:**
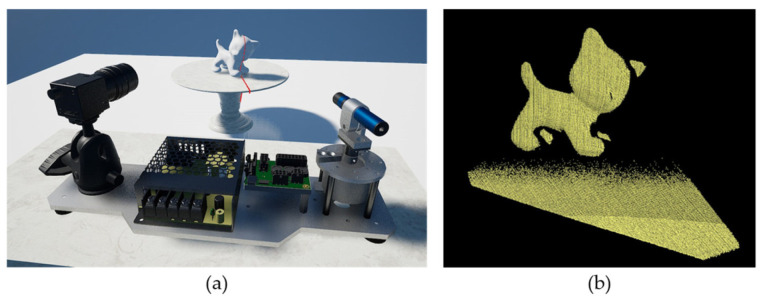
Three dimensional (3D) reconstruction using our system, (**a**) setup of the system, (**b**) reconstruction results.

In this study, an LT system with a rotating line laser and wide-angle camera was proposed as shown in Figure 1. We only rotate the line laser, not the entire system, to scan a wide area. Rotating a laser is easy because the line laser is light, leading to a simple system. We do not need to rotate the camera because it already has a wide field of view with a proper lens (e.g., fish-eye lens).

With this setup, the first step is to precisely estimate this rotation axis with respect to the camera coordinates, to measure the depth of an object using triangulation [23]. Owing to various reasons, such as the fabrication error of mechanical parts, line laser misalignment to the rotation axis, line laser optical properties, and so on, the rotation axis of the line laser is somewhat different from the ideal CAD design. However, this axis is precisely measured to obtain accurate 3D information from the LT method. In this paper, we propose a novel calibration between the camera coordinate system and the rotating line laser coordinate system using a cone model. Finding the axis of rotation with respect to the camera coordinate system is the key for accurate calibration. We noticed that the cone shape is always created while the plane of the line laser rotates about the rotation axis of the motor due to inevitable imperfect alignment between the line laser and the rotating axis. Therefore, we estimate the axis of rotation using the cone model because the central axis of the cone and the rotation axis of the motor are same. The cone model enables us to estimate the rotation axis accurately leading to improved calibration results. In summary, our contribution is to propose a cone model for an accurate calibration between the camera and the rotating line laser resulting in an accurate and wide 3D scanning system.

The rest of Section 1 defines the notation and coordinate system, and Section 2 introduces the schematic diagram of the system and the calibration process. In Section 3, the triangulation method through the line-plane intersection is described, and the calibration between the fixed line laser and camera, and the calibration between the rotating line laser and camera are explained. In Section 4, the calibration accuracy according to the rotation axis of the line laser is evaluated, and the 3D reconstruction results of the RGB-D camera and this system are compared. Then, the accuracy of the 3D depth estimation is analyzed using a planar model. Finally, the conclusions are presented in Section 5.

## 2. Overall System

### 2.1. Hardware

**Figure 2:**
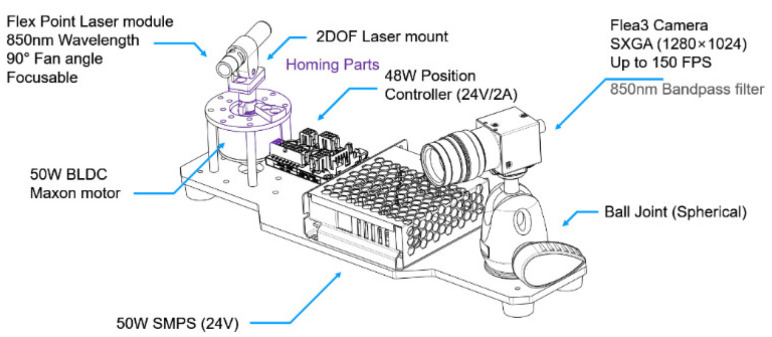
Scanning system with a line laser fixed to a motor and infrared camera.

Our scanning system has a line laser fixed to a motor and a camera with an infrared (IR) filter, as shown in Figure 2. The line laser used in our system has 90° fan angles at 850 nm wavelength (FLEXPOINT MVnano, Laser Components, Bedford, NH, USA). We designed a mount that can fix and adjust the orientation of the line laser. This mount has two degrees of freedom rotating about pitch and roll axes. We will explain the effect of the roll angle with simulation. Our brushless direct current (BLDC) motor with an incremental encoder has approximately a resolution of 0.08°. To obtain the absolute angle θ about the motor axis, we calculated the angle with the incremental encoder from the home position. We used a camera with a wide-angle lens and an 850 nm infrared bandpass filter to detect only IR light (FLEA3, FLIR System, Wilsonville, OR, USA). The camera with a lens has vertical and horizontal field of view of approximately 57° and 44°, respectively. The camera resolution is 1280 by 1080 and its acquisition speed is 150 frames per second.

### 2.2. Process of Scanning System

**Figure 3:**
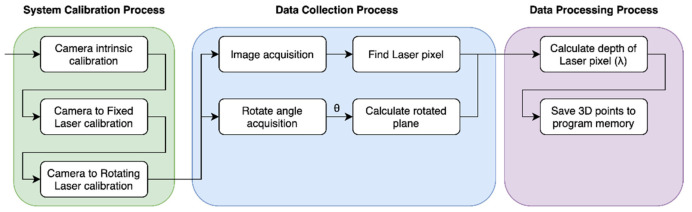
Flowchart of our scanning process.

As shown in Figure 3, we executed three processes to acquire 3D scan data. Because our scanning system consists of three components, we determined the transformation relationship between the camera and other devices via calibration methods.

Because every point of the 3D scan data was acquired from the image, and the area illuminated by the laser was narrow in the field of view of the sensor, we rotated the laser via the motor and recorded a camera image for every angle of the motor. By using calibration data and the angle value of the motor, we calculated 3D points from each recorded image. The 3D points are stored in the program memory, and we can visualize or store them in the storage of our PC.

### 2.3. Camera Calibration

In digital imaging, the camera is modeled as a pinhole camera model. The pinhole camera model is a method for projecting any single point present in a 3D space into image coordinates (r, c). It consists of a combination of intrinsic parameters $\left(f_{x},\;f_{y},\;c_{x},\;c_{y},\;\alpha \right)$ and extrinsic parameters, rigid body transform to a world coordinate system.

The parameters estimated through the camera calibration process are used. Currently, most intrinsic and extrinsic parameters are estimated using a checkerboard printed on a flat plane [24]. If we know the intrinsic parameters of the camera, we can calculate where any points represented in the camera reference frame are projected into the image coordinate system. Furthermore, knowing the 3D point to 2D point corresponding pairs of more than four points with the camera’s intrinsic parameters identified, we can estimate the rigid body transform of it with respect to the camera coordinate system.

**Figure 4:**
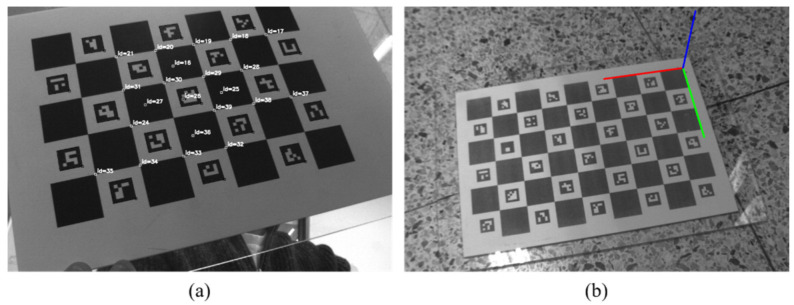
(**a**) Intrinsic calibration and (**b**) Extrinsic calibration.

For the experiments, we used a checkerboard with an AR marker, ArUco (ChArUco), to estimate the rigid body transform, as shown in Figure 4. Its parameters were also used to identify the laser plane in the camera reference coordinate system.

## 3. Solving Extrinsic Calibration

Our scanning system estimates 3D depth information based on the triangulation of pixel points in the image coordinate system. Therefore, it is necessary to know the ray information of the camera coordinate system and the plane information of the line laser, such as the normal vectors, to calculate the depth. Our scanning system uses a rotating line laser and fixed camera. The first step is to perform a calibration that calculates the geometrical relationship between the camera and the laser-rotating axis. We performed this calibration using a cone shape model because a cone is made from multiple planes generated by the rotation of the line laser. In the following subsections, we explain this process in detail. First, the triangulation method is introduced, and then the process of obtaining the relationship between the camera and plane generated by the line laser is explained. After that, the calibration to find the transformation between the rotation axis of the line laser and the camera is explicated. Finally, we obtained the depth map from the encoder value of the motor and the information between the line laser and camera.

### 3.1. Triangulation

**Figure 5:**
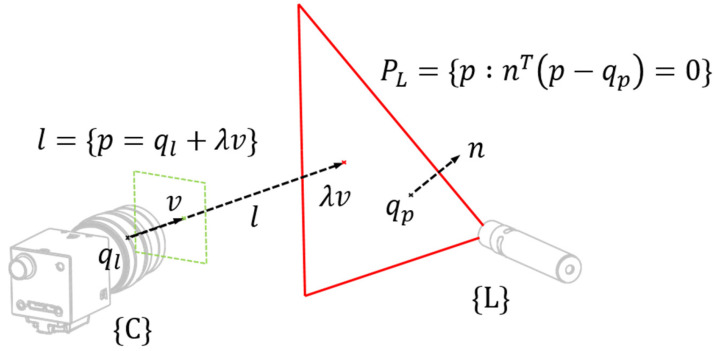
Line-Plane intersection.

Triangulation refers to the process of calculating the depth value λ of a common point observed by two sensors, provided that a transformation matrix between both systems, such as camera and line laser is known. In 3D space, a common point is created through line-line or line-plane intersections. The line l is the ray of the camera, and the plane $P_{L}$ is the line laser plane. Our system corresponds to a line-plane intersection as shown in Figure 5. The intersection between a plane and a line is given by

(1) $$\lambda =\frac{n^{T}\left(q_{p}-q_{L}\right)}{n^{T}v},$$

where n, $q_{p}$, $q_{L}$ and v are the normal vector of $P_{L}$, a point of $P_{L}$, a point and a directional vector of l, respectively.

$q_{L}$ becomes a zero vector when passing through the origin of the camera coordinate system. Therefore, the depth λ can be calculated as

(2) $$\lambda =\frac{n^{T}q_{p}}{n^{T}v}.$$

### 3.2. Camera to Fixed Laser Calibration

**Figure 6:**
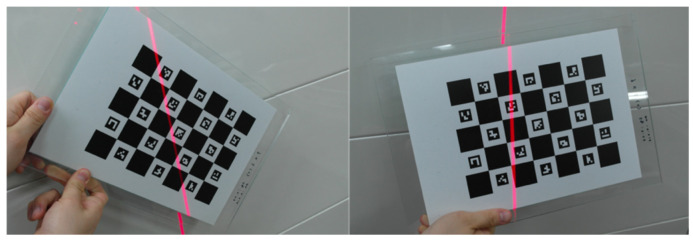
Calibration of laser plane with respect to camera coordinate.

To calculate the relationship between fixed line laser and the camera, point p on the plane $P_{L}$ acquired from multiple poses are required, as indicated in Figure 6. Because the transformation matrix TWC  from camera (C) coordinate to world (W) coordinate can be acquired through the AR marker, the laser points on the checkerboard can estimate the depth λ through triangulation. Point p on the estimated plane $P_{L}$ can be represented by the following implicit equation:

(3) $$n^{T}\left(p-q\right)=0.$$

The elements $p_{1},\;p_{2},\cdots ,p_{n}$ of the points set {p} collected from n poses and the plane $P_{L}$ were formulated with matrix multiplication as follows.

(4) [p  1T−1p  2T−1⋮⋮p  nT−1][nqTn]=0.

Because the set of points {p} acquired through the camera inevitably contains noise data, this equation is not equal to zero. The solution can be found by the linear least-squares method. Therefore, the equation of the plane $P_{L}$ generated by the line laser can be estimated with respect to the camera coordinates. In addition, we normalized the normal vector n of the plane to simplify the geometric analysis in the next section.

### 3.3. Camera to Rotating Laser Calibration

When the line laser rotates along the rotation axis of the motor, the plane created by the line laser rotates along the rotation axis. This means that the equation of the plane $P_{L}$ generated by the line laser in the camera coordinate also changed through an angle θ. The equation of $P_{L}$ is related to the rotation axis ω and the translation vector tLC   of the line laser expressed in the camera coordinates. The angle θ can be obtained through an incremental encoder with the motor. If the line laser is fixed, the parameters (n, q) for the equation of the plane $P_{L}$ are constant. However, in our system, the equation of the plane $P_{L}$ depends on the parameters tLC  , ω and θ because the line laser rotates along the rotation axis of the motor.

#### 3.3.1. Find a Point on Rotating Axis

To estimate the translation vector tLC  , the various planes $P_{L}{}_{\theta }$ rotated around angle θ should be calculated. The implicit equation of plane sets with m poses can be expressed using Equation (5).

PL1: n  1T(p−q  1 )=0PL2: n  2T(p−q  2 )=0⋮PLm: n  mT(p−q  m )=0

(5) PL1∩PL2∩⋯∩PLm=tLC.

**Figure 7:**
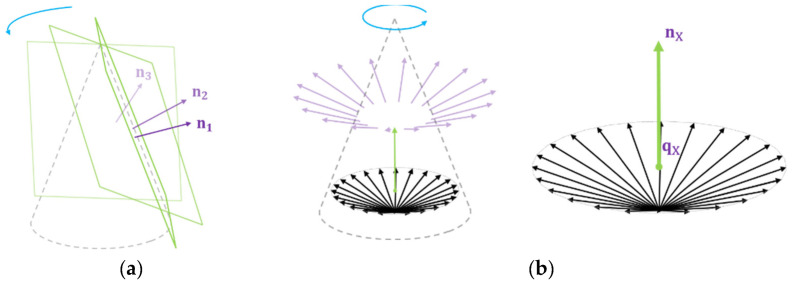
(**a**) The laser plane with an angle to the rotating axis is forming a cone. (**b**) The trace of the normal vector of the rotating plane is forming an inverted cone after starting points of them are moved to a point on the rotating axis.

If a plane rotates along an arbitrary axis in a 3D space, there exists an intersection point through which all planes pass through, excluding a specific case (Figure 7a). We discuss this specific case with the simulation in Section 4.1. Simultaneously, the intersection point tLC exists on the rotation axis of the motor. Because the parameters $n_{1\ldots m},\;q_{1\ldots m}$ about these planes $P_{L_{1\ldots m}}$ had noise data, we obtained the position vector tLC using the least squared method, as represented by Equation (6).

(6) Ax=[n  1T−  n1Tq1n  2T−  n2Tq2⋮⋮n  mT−  nmTqm][p1]≈0.

This position vector p corresponds to the translation tLC from the camera coordinate to the laser coordinate. In addition, Equation 6 can be interpreted as a geometric meaning that minimizes the distance to a common point for all planes. The shortest distance from a point to the plane was along a line perpendicular to the plane.

(7) $$d=\frac{\left|n^{T}\left(p-q\right)\right|}{\|n{\|}_{2}}=\left|n^{T}\left(p-q\right)\right|\;\;(∵{\left\|n\right\|}_{2}=1).$$

#### 3.3.2. Find Rotating Orientation

Similar to estimating the translation of the rotation axis, we used $\left\{P_{L}\right\}$ to estimate the direction of ω. The parameters constituting each equation of $P_{L}$ equation are (n, q). n is a normal vector of the plane $P_{L}$ and q is a point on the plane. Because n is a free vector, the starting point of the vector can be freely moved. If the starting points of the normal vectors move to a point on the rotating axis, the moved normal vectors form an inverted cone shape. The inverted cone shape is shown in Figure 7b.

As shown in Figure 7b, one end of the normal vector points toward the origin, and the other end points to the base of a cone in 3D space. The shape of the cone is deformed according to the position of the axis of rotation and the position of the laser, but the central axis of the cone and the axis of rotation of the motor are always the same.

The central axis of the cone always is parallel with the normal vector of the base of the cone. Finding the normal vector of the base of the cone can be regarded as the same task as finding the direction vector of the motor’s axis of rotation. Let us denote this base plane of the inverted cone as $P_{X}$ with n  XT(p  −q  X )=0.

The process of finding the rotation axis direction from the collected plane $\left\{P_{L}\right\}$ is as follows: We generate a matrix consisting of n corresponding to the normal vector of the collected planes $\left(P_{L}{}_{1},\;P_{L}{}_{2},\;\ldots ,\;P_{L}{}_{m}\right).$ Each row of a matrix consisting of n refers to a point on the base $\left(P_{X}\right)$ of the cone, so it satisfies the plane equation. Because we have m normal vectors, this can be expressed as follows:

PL1: n  XT(n  1 −q  X )=0

PL2: n  XT(n  2 −q  X )=0⋮

PLm: n  XT(n  m −q  X )=0.

In the following matrix form, it becomes:

(8) [n  1T−1n  2T−1⋮⋮n  mT−1][n  X q  XTn  X ]=0.

Because noise exists in the data, the expression is modified in an approximate form, and an optimal solution is obtained in the form of Ax≈0 with constraint $\left|n_{X}^{\;}\right|=1.$

### 3.4. Rotation of Line Laser Plane around an Axis

**Figure 8:**
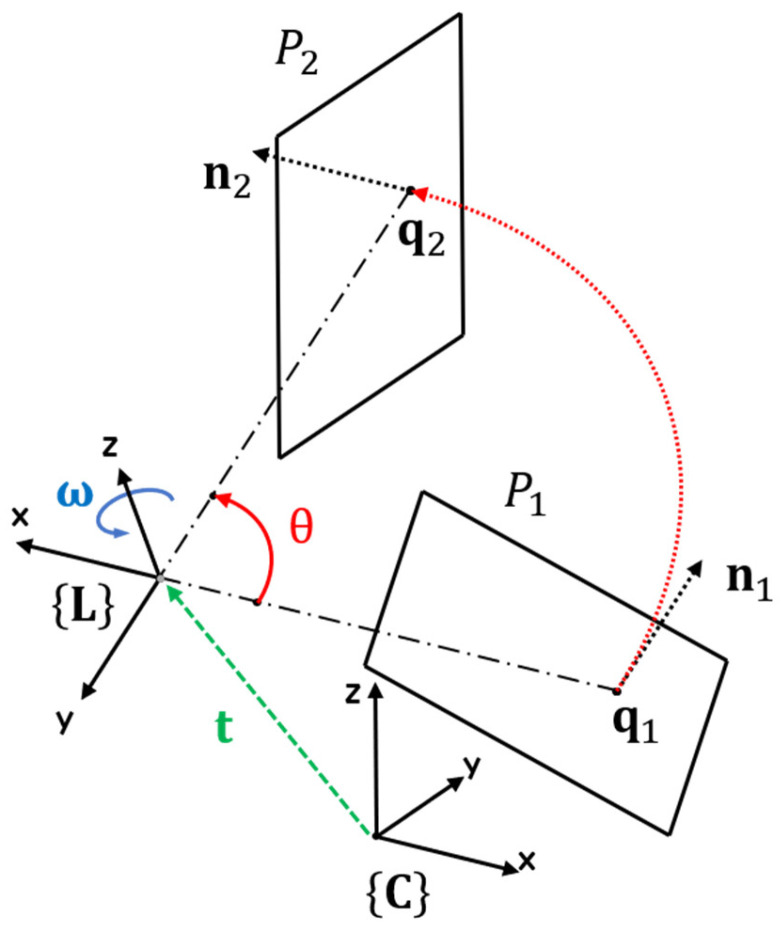
The shape and trajectory of the plane rotating by θ along arbitrary axis (red).

The plane created when the line laser rotates can also be determined through the transformation matrix between the camera and the rotation axis. The rotated line laser plane about the axis should be calculated because it is difficult to calibrate between the camera and line laser plane whenever the motor rotates by θ. As shown in Figure 8, plane $P_{2}$ is a new plane created when plane $P_{1}$ rotates about an arbitrary axis of rotation. The plane P is expressed as a normal vector **n** and a point q on the plane. Therefore, both the normal vector $n_{1}$ and point $q_{1}$ on plane $P_{1}$ need to be rotated about the Z-axis of the laser coordinate (i.e., rotation axis of the motor). A vector in ${\mathbb{R}}^{3}$ can be rotated by Rodrigues’ rotation formula. By applying the formula to q  1 and $n_{1}^{\;}$, we find the plane rotated by θ as follows:

q2 =R  ω (θ)(q1 −t)+t,n2 =R  ω (θ)n1 ,

where

$$R_{\omega }\left(\theta \right)=I+\left(\sin\theta \right)\Omega +\left(1-\cos\theta \right)\Omega^{2}\;\mathrm{and}\;\Omega =\left[\begin{array}{ccc} 0 & -\omega_{z} & \omega_{y}\\ \omega_{z} & 0 & -\omega_{x}\\ -\omega_{y} & \omega_{x} & 0 \end{array}\right].$$

## 4. Results

To evaluate the calibration described in the previous section, we performed simulations and experiments. First, we evaluated the calibration accuracy in the simulation with respect to the change in α, the angle between the rotation axis of the line laser and the line laser plane. Then, we presented two experimental results in a real environment. For comparison, we used a commercial depth camera (RealSense SR305, Intel, Santa Clara, CA, USA), which has a relatively high-quality in the short range. In the first experiment, a plane checkerboard was scanned and then we measured how far the set of points was from that plane. In the second experiment, we show the scan results for various objects using two scan systems.

### 4.1. Simulation

**Figure 9:**
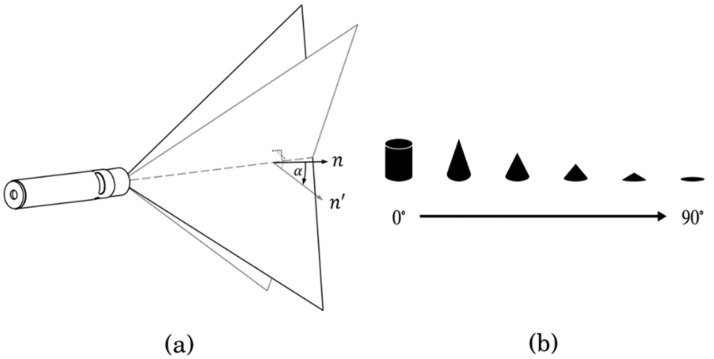
Simulation condition (**a**) Angle α between the axis of rotation and the normal vector of the plane generated by the line laser and (**b**) the change of the shape as the angle α increases.

To obtain a depth map for the camera view, we rotated the line laser about a rotation axis. As shown in Figure 9, α  determines the 3D shape that is tangential to the collected laser planes. The type of this tangential shape is either cone or cylindrical if α is less than 90°.

As the value of α approaches 0°, the slope of the cone becomes steep. Therefore, if the base area of the cone is constant, the intersection point increases as the value of α approaches 0°. If the value of α is 0°, the slope of the cone becomes +∞. This means that the intersection point cannot be determined. For this reason, the calibration method for models with very high slope values, such as cylinders, was prepared separately.

**Figure 10:**
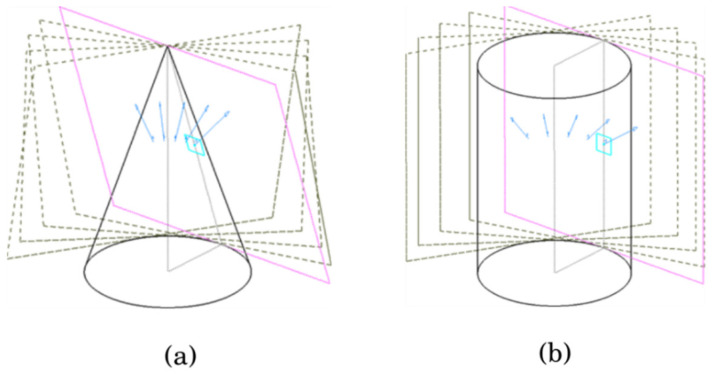
A different shape of the set of planes, (**a**) Cone shape, (**b**) Cylinder shape.

If the angle α becomes 0°, it shows a cylindrical shape instead of a cone shape, while the line laser rotates about the axis of rotation. If it is formed in a cylindrical shape, as shown in Figure 10b, the following method is used. Unlike the cone shape, normal vectors of planes tangent to a cylinder are in a 2D space perpendicular to the axis of rotation. We find the intersected lines between the collected planes and the plane of the normal vectors. Principal component analysis (PCA) was used to estimate the plane of normal vectors. The center of the circle was estimated using these intersection lines. The distance between the center of the circle and the angle bisector of any two outer tangents was zero. Because of noise, we found a point where the value of the distance to the bisector was minimal from multiple intersections. This point was considered as the coordinate passing through the central axis of the cylinder.

**Figure 11:**
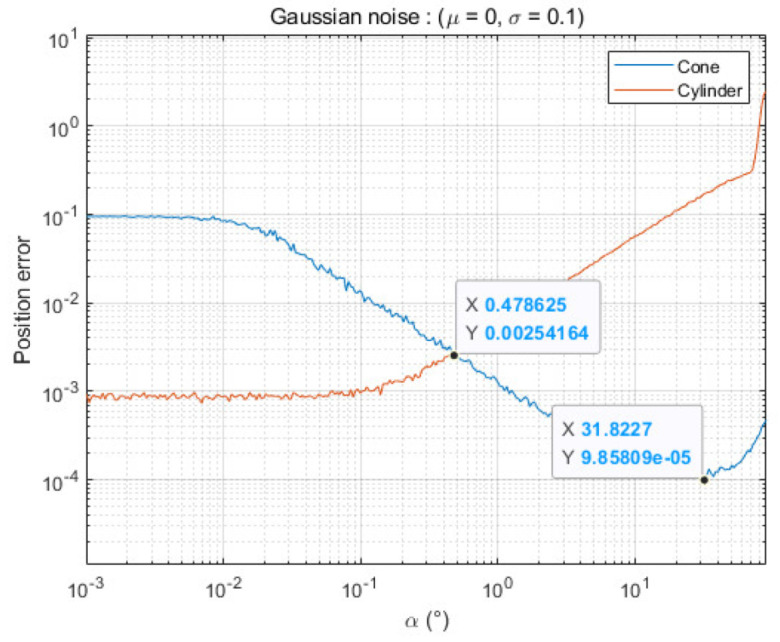
Results of the simulation according to the angle α.

To correctly project in 2D using PCA, the normal vector of the tangent plane must be in a 2D space. That is, it works correctly only for special cases where α=0°. Nevertheless, it is also applicable for otherwise (α≠0°), but it will serve as an error for estimating the rotational axis. For this reason, we can see that the estimation error increases as the value of α increases, as shown in Figure 11.

Although the points on the plane generated by the line laser in camera coordinates were estimated through the detection of the checkerboard and line laser in the real experiment, we assumed that the points were given values in the simulation. In addition, we added Gaussian noise with a standard deviation of 0.1 mm to emulate the noise of the real environment in the simulation environment.

Because the ground truth is known in the case of simulation, unlike actual experiments, an accurate calibration difference can be obtained. To verify the accuracy of the calibration according to α, the distance $e_{p_{1}}$ between the estimated point (cone apex) and the true rotation axis of the line laser was calculated using different angles α. 

Simulation results show that if the angle α is more than 0.47°, a cone-shape-based method is better than a cylinder-based method (Figure 11). This means that the cylinder model is fine if we can adjust the angle between the rotation axis and the laser plane precisely; however, if there is an error of more than 1°, our cone model leads to better results. Furthermore, the results also show that the lowest estimation error occurs at an angle α=31.82° under our noise model. For this reason, we adjusted the angle α of our scanning system in real experiments to be approximately 30°.

### 4.2. Real Experiments

In this experiment, we compared our system with the SR305 camera, which has a relatively high precision compared to other RGB-D cameras in terms of the performance of the depth map. Two experiments were performed. During the experiments, we fixed the two scanning systems together at the plate. First, we evaluated the planarity of the plane, which was estimated for the checkerboard after calibration between the line laser and camera. We also compared the quality of the results scanning various objects, including the environments.

#### 4.2.1. Planarity of the Plane

Because of the flatness condition, the checkerboard is suitable for evaluating the performance of depth estimation. Moreover, we can easily define the reference coordinate with respect to the camera coordinates using the pattern of the checkerboard. Because the origin of the scanning system also has the camera coordinates, it is easy to calculate the error of the planarity. Therefore, we estimated the depth map using the checkerboard plane using two scanning systems. We removed the outlier points in the result using a point cloud tool, such as MeshLab, because it is not easy to only have the checkerboard in the field of view. To evaluate the accuracy of the planarity, each camera estimated world coordinates using intrinsic parameters. We calculated the distance $e_{p_{2}}$ between the reference frame and the estimated points on the checkerboard plane using Equation (9). The parameters n, q of the plane can be obtained using the perspective-n-point algorithm and point vectors p are achieved by depth estimation using the scanning systems.

(9) $$e_{p_{2}}=\frac{\left|n^{T}\left(p-q\right)\right|}{{\left\|n\right\|}_{2}}$$

**Figure 12:**
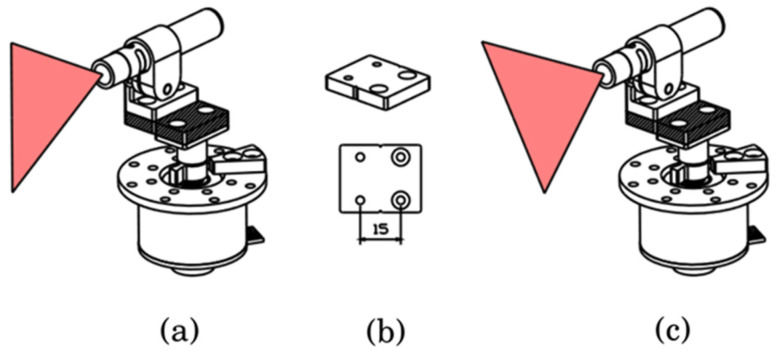
Experiment setup, (**a**) ill-posed condition ($\alpha_{ill}$, (**b**) displacement part, and (**c**) well-posed condition ($\alpha_{well}$).

**Figure 13:**
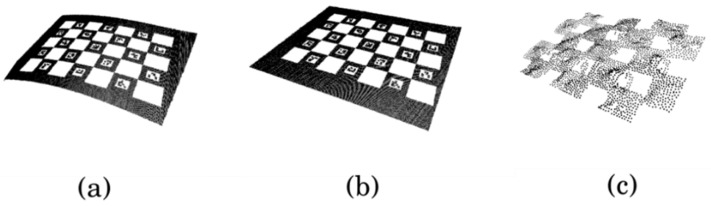
Results of 3D reconstruction: (**a**) Our system at $\alpha_{ill}$, (**b**) $\;\alpha_{well}$ and (**c**) SR305.

**Figure 14:**
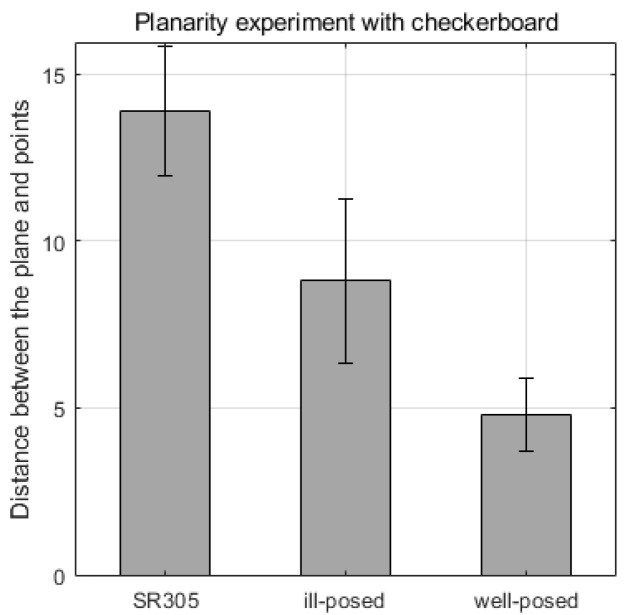
Distance errors from the checkerboard plane to every point.

Meanwhile, to verify the effect of the angle α of the rotation axis and the line laser as assessed in the simulation, we experimented with $\alpha_{ill}$ and $\alpha_{well}$ as shown in Figure 12a,c. As shown in Figure 12b, the line laser was moved 15 mm from the axis of rotation. The reason is to easily check the difference in accuracy according to α, and to confirm the robustness of the calibration estimation for design errors. As shown in Figure 13, the results of the 3D reconstruction of the checkerboard are indicated by each method. The average and standard deviation of the distances $e_{p_{2}}$ between the points from the plane are shown in Figure 14. We also compared the performance of the 3D reconstruction between our system and SR305 on Table 1. Since our system can detect relatively large distances, the scan volume size of our system is far bigger than SR305.

#### 4.2.2. Scene Reconstruction

**Figure 15:**
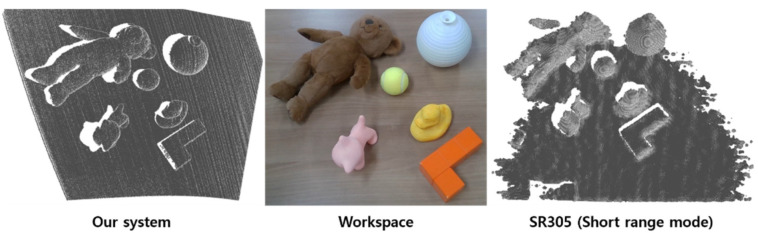
Three dimensional reconstruction of multiple objects with background.

**Figure 16:**
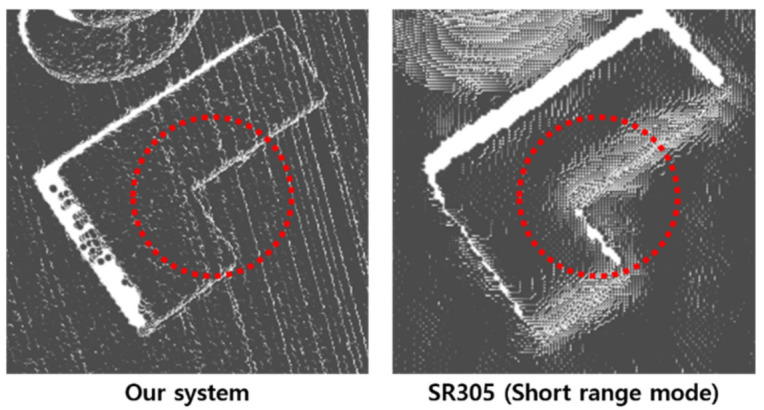
Comparison of the result that was scanned a cube object using two devices.

Unlike previous quantitative assessments, we verified additional qualitative results regarding the reconstruction of various shapes of objects using two scanner systems. As shown in Figure 15, we scanned multiple hand-held items, such as a tennis ball and a teddy bear. Among the scanned objects, the result of the orange cube with sharp edges was significantly different between the two sensors, as depicted in Figure 16.

**Figure 17:**
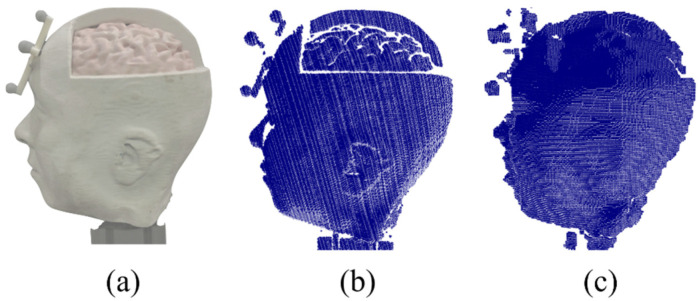
Comparison of the 3D reconstruction of a human plaster: (**a**) Original, (**b**) our system and (**c**) SR305.

As depicted in Figure 17a, a human plaster with complicated features is appropriate for a qualitative comparison. The size of the plaster was similar to that of the human head. Compared with Figure 17b,c, the cortex areas of the results that were scanned by the two scanners were different. Unlike SR305, our method relatively measured the curvature components of the object.

## 5. Conclusions

We developed a 3D scanning system for a broad region using a rotating line laser and camera. The transformation should be acquired between the camera coordinates and the plane of the line laser to reconstruct an object. The information about the plane of the line laser with respect to the camera coordinate whenever the plane is rotated should be determined for 3D reconstruction. In this study, we proposed a novel calibration method to determine the relationship between the rotation axis of the line laser and the camera. The center of a cone or cylinder shape generated while the line laser was rotating was calculated. In the simulation study, we evaluated the accuracy of the proposed calibration using two models. The simulation shows that the proposed cone model is superior to the simple cylinder model if the angle alignment error is greater than approximately 0.5°. This means that, in most cases, we have better results when using the proposed cone model because the misalignment of the laser plane and the rotation axis is larger than this value. Finally, we evaluated the performance of the 3D reconstruction of our system through real experiments and compared the results with those of a commercial RGB-D camera. The quality of the restoration using our system was far superior to that of the RGB-D camera. Our system can acquire the depth map of object at a far distance compared to the RGB-D camera. Our future work will include multiple line laser calibration for improving scanning speed and 3D object recognition using this improved depth information. 

## Figures and Tables

**Table 1:** Comparison between our system and the commercial sensor (SR305).

	SR305	Our System
Estimated accuracy [mm]	13.8 ± 3.8	4.8 ± 2.2
Working distance [m]	0.2~1.5	0.5~3.0
Pyramid volume [m^3^]	1.8838	7.1297
Relative pointcloud density	×1	×2.786
Minimal components	IR Laser Projector IR Camera	IR Line Laser IR Camera Motor assembled encoder
